# ATR- and ATM-Mediated DNA Damage Response Is Dependent on Excision Repair Assembly during G1 but Not in S Phase of Cell Cycle

**DOI:** 10.1371/journal.pone.0159344

**Published:** 2016-07-21

**Authors:** Alo Ray, Chessica Blevins, Gulzar Wani, Altaf A. Wani

**Affiliations:** 1 Department of Radiology, The Ohio State University, Columbus, Ohio, 43210, United States of America; 2 Department of Radiology, Department of Molecular and Cellular Biochemistry, James Cancer Hospital and Solove Research Institute, The Ohio State University, Columbus, Ohio, 43210, United States of America; The University of Hong Kong, HONG KONG

## Abstract

Cell cycle checkpoint is mediated by ATR and ATM kinases, as a prompt early response to a variety of DNA insults, and culminates in a highly orchestrated signal transduction cascade. Previously, we defined the regulatory role of nucleotide excision repair (NER) factors, DDB2 and XPC, in checkpoint and ATR/ATM-dependent repair pathway *via* ATR and ATM phosphorylation and recruitment to ultraviolet radiation (UVR)-induced damage sites. Here, we have dissected the molecular mechanisms of DDB2- and XPC- mediated regulation of ATR and ATM recruitment and activation upon UVR exposures. We show that the ATR and ATM activation and accumulation to UVR-induced damage not only depends on DDB2 and XPC, but also on the NER protein XPA, suggesting that the assembly of an active NER complex is essential for ATR and ATM recruitment. ATR and ATM localization and H2AX phosphorylation at the lesion sites occur as early as ten minutes in asynchronous as well as G1 arrested cells, showing that repair and checkpoint-mediated by ATR and ATM starts early upon UV irradiation. Moreover, our results demonstrated that ATR and ATM recruitment and H2AX phosphorylation are dependent on NER proteins in G1 phase, but not in S phase. We reasoned that in G1 the UVR-induced ssDNA gaps or processed ssDNA, and the bound NER complex promote ATR and ATM recruitment. In S phase, when the UV lesions result in stalled replication forks with long single-stranded DNA, ATR and ATM recruitment to these sites is regulated by different sets of proteins. Taken together, these results provide evidence that UVR-induced ATR and ATM recruitment and activation differ in G1 and S phases due to the existence of distinct types of DNA lesions, which promote assembly of different proteins involved in the process of DNA repair and checkpoint activation.

## Introduction

In response to DNA damage, living cells arrest at discrete phases of the cell cycle either to allow DNA repair which is essential for cell survival or if the damage is too high promote cell death [1;2]. The mammalian nucleotide excision repair (NER) pathway removes a wide range of chemically and conformationally diverse DNA adducts, including ultraviolet radiation (UVR)-induced bulky DNA adducts, e.g., cyclobutane pyrimidine dimers (CPD) and pyrimidine (6–4) pyrimidone photoproducts (6-4PP) [3]. One sub-pathway of NER, global genomic NER (GG-NER), removes DNA damage from the entire genome whereas DNA lesions in the transcribed strand of active genes are preferentially eliminated by transcription-coupled NER (TC-NER) [4]. In GG-NER, damage is recognized by the DDB (damaged DNA binding protein), involving DDB1 and DDB2, and XPC (Xeroderma pigmentosum complementation group C)-RAD23B complexes [5;6]. The DDB complex initially recognizes the CPD lesions and helps in recruiting XPC, whereas 6-4PP lesions are directly recognized by XPC independent of DDB [5–8]. The DDB1-CUL4-ROC1 complex associates with DDB2 adapter and Cullin 4A-mediated proteolysis of DDB2 at the DNA damage sites regulates the lesion recognition by XPC [9]. Cullin 4A also ubiquitylates XPC, which mediates DNA binding by XPC [10]. In turn, XPC orchestrates the sequential recruiting of factors of multi-protein NER complex including XPA, XPG, and TFIIH components that enable opening of the DNA helix around the damage site to form a bubble [7]. XPA stabilizes the bubble and helps in positioning XPF and XPG endonucleases for respective 5′ and 3′ incisions to excise out a 24–32 bp oligonucleotide containing damaged lesion. The resulting short ssDNA gap is filled by repair synthesis, and finally the nick is ligated to complete NER [3;11]. In TC-NER, damage is recognized by CSA and CSB which help in subsequent recruitment of XPA and other NER proteins. Therefore, XPA is an integral component of DNA damage processing by both GG-NER and TC-NER.

Cellular response to DNA damage is controlled by the phosphoinositide-3-kinase-related-protein kinase (PIKK) family including ATR (Ataxia telangiectasia- and Rad3- related) and ATM (Ataxia telangiectasia mutated) kinases [12;13]. Seckel (ATR-defective) and A-T (ATM-deficient) cells exhibit impaired signaling due to the defects in repair and checkpoint activation. Several studies implicated that short ssDNA (single-stranded DNA) gaps caused by UV damage results in activation of ATR-dependent repair and checkpoint pathways [14–16]. In addition, during S phase, replication forks encounter the CPD and 6-4PP lesions that provoke stalling of the replication forks at the single-strand breaks (SSBs). These breaks are processed to long ssDNA, where RPA binds and initiates the recruitment of a complex array of DNA damage response (DDR) proteins, including ATRIP-ATR complex, TopBP1, MRE11, Rad50, Rad17, and 9-1-1 complex [17–19]. If unresolved, stalled replication forks collapse and lead to the formation of DSBs. The Mre11/Rad50/Nbs1 (MRN) complex recognizes the resulting DSBs and facilitates ATM recruitment and activation [20–23]. The MRN complex, together with CtIP, possesses 3′-to-5′ exonuclease and endonuclease activities that initiate the resection of DSB ends [20]. The resulting ssDNA tails search for homology and invade the sister chromatid for homologous recombination (HR)-mediated repair [24–26].

Activated ATR and ATM phosphorylate numerous DNA damage response and repair proteins at single or multiple Ser/Thr-Gln (S/T-Q) sites. ATR predominantly phosphorylates Chk1 and ATM phosphorylates Chk2, which result in checkpoint activation. Activated Chk1 and Chk2 phosphorylate Cdc25 phosphatases to inhibit their function, and the cells delay progression through the cell cycle [27;28]. ATR and ATM also phosphorylate histone H2AX at Ser-139 (γH2AX) in response to DNA double-strand breaks (DSBs), which spreads along the DNA up to 200–400 kb and helps in the recruitment of proteins involved in DNA damage repair and checkpoint activation [29]. Activated ATR and ATM also phosphorylate BRCA1, which is required for S and G2/M phase checkpoints and HR-mediated repair during S and G2 phases [30;31]. Taken together, the signals emanating from the active ATR and ATM checkpoint complex result in cell cycle checkpoint arrest, inhibit DNA replication, stabilize stalled forks, and initiate DNA repair of damaged substrates.

While ATR- and ATM-mediated signaling pathways have been systematically investigated, the mechanisms of DNA lesion recognition by ATR and ATM upon UV damage are still unclear. Previous studies support that ATR is predominantly responsible for DDR upon UVR exposure, and H2AX is phosphorylated both in G1 and S phases by ATR in response to UV irradiation [26;32–38]. Recently, we and others have shown that UVR-induced DNA damage and replication stress also activates ATM [39–42], which in turn phosphorylates H2AX [40;41]. Moreover, we showed that DDB2 and XPC influence ATR and ATM recruitment to the damage site and promote their activation by phosphorylation [41]. Although all these studies support that ATR and ATM play a role in UV damage response, these studies did not assess how and when ATR and ATM are recruited to the damage sites, whether ATR and ATM recruitment requires the assembly of a NER pre-incision complex, and if it is influenced by different kinds of DNA lesions generated during G1 and S phase of the cell cycle. To gain an in-depth insight into the intricate molecular mechanism(s) of ATR and ATM recruitment to different types of UVR-induced lesions, we investigated (i) the time of recruitment, (ii) the role of DDB2 and XPC downstream factor XPA that is required for both GGR and TCR pathways, and (iii) the dependence on NER proteins during G1 and S phases of cell cycle. We show that ATR and ATM are promptly recruited to DNA damage sites following cellular UVR exposure resulting in early γH2AX phosphorylation, and their recruitment is dependent not only on DDB2 and XPC, but also on XPA. Our further investigation revealed that ATR and ATM recruitment and H2AX phosphorylation are dependent on DDB2, XPC, and XPA during G1 phase when only the UV lesions are present. In contrast, during S phase when the UVR lesions predominantly result in stalled replication forks, ATR and ATM recruitment and H2AX phosphorylation is not dependent on DDB2, XPC, and XPA proteins. These results provide key mechanistic details of ATR and ATM recruitment and the nature of lesion processing and checkpoint signaling events following UVR exposures.

## Materials and Methods

### Cell lines and antibodies

XP-E (GM01389, DDB2-mutated), XP-C (GM02096, XPC-mutated), XP-A (GM05509, XPA mutated) cells were from ATCC. OSU-2 (Normal human fibroblasts or NHF) was generated in our laboratory [43]. The cells were cultured as described [44]. XPC, DDB2, CPD, antibodies were raised in our laboratory. Antibodies specific for pATR (Ser 428), pATM (Ser 1981), pChk2 (Thr68), pChk1 (Ser 296), γ-H2AX (Ser139), Chk1 (2345), and Chk2 (2662) were from Cell Signaling Technology. H2AX (sc-54606), ATM (sc-23921) and ATR (N-19) (sc-1887), antibodies were from Santa Cruz Biotechnology. Alexa Fluor 488 dye (A-11094) and Texas Red conjugate (T-2767) secondary antibodies were from Invitrogen. Goat anti-rabbit IgG IR Dye 800CW (926–32211) was from LI-COR biosciences.

### UV irradiation, protein isolation, and Western blotting

These were performed as described in [45]. Cells were washed with phosphate-buffered saline (PBS) and irradiated through a germicidal lamp (254 nM) at a dose rate of 1.0 J m^2^/s as measured with a Kettering model 65 radiometer (Cole-Palmer, Vernon Hills, IL, USA). Media was added to the cells, returned to 37°C incubator to allow repair and harvested at the indicated post-UV irradiation times. Total protein was extracted from the cells using sodium dodecyl sulfate (SDS) lysis buffer (62 mM Tris–HCl, pH 6.8, 2% SDS, 10% glycerol) with protease and phosphatase inhibitors followed by boiling for 8 min. Protein amount was estimated using Bio-Rad DCTM Protein assay kit, and the whole cell lysates were resolved by SDS–polyacrylamide gel electrophoresis (PAGE) using Novex Tris-Glycine gels (Invitrogen, Carlsbad, CA, USA) followed by Western blotting to detect specific proteins.

### Isolation of G1 and S phase cells

Cells were arrested in G1 by serum starvation for 48 h. The S phase cells were detected by EdU labeling as described in [46;47]. EdU was purchased from Invitrogen. Cells were incubated with serum-free DMEM supplemented with 10 μM EdU for 3 h after the UV irradiation. Cells were then washed with PBS, followed by fixation, permeabilization, and fluorescent labeling with Alexa Fluor 488 dye.

### Immunofluorescence

Immunofluorescence staining was conducted essentially as previously described [41;45]. Cells were cultured on coverslips, covered with a 5-um isopore polycarbonate filter, UVC-irradiated (100 J/m2) followed by post-UVR incubation times. Cells were then washed with PBS, followed by fixation and permeabilization. Next, cells were stained with appropriate primary antibodies followed by fluorescein isothiocyanate (FITC) or Alexa Fluor 488 and Texas Red-conjugated secondary antibodies. The intensity of nuclear fluorescence was measured using Nikon fluorescent microscope E80i (Tokyo Japan) and processed with SPOT software.

## Results

### ATR and ATM are rapidly recruited to UVR damage sites and localize with XPC

**Fig 1:**
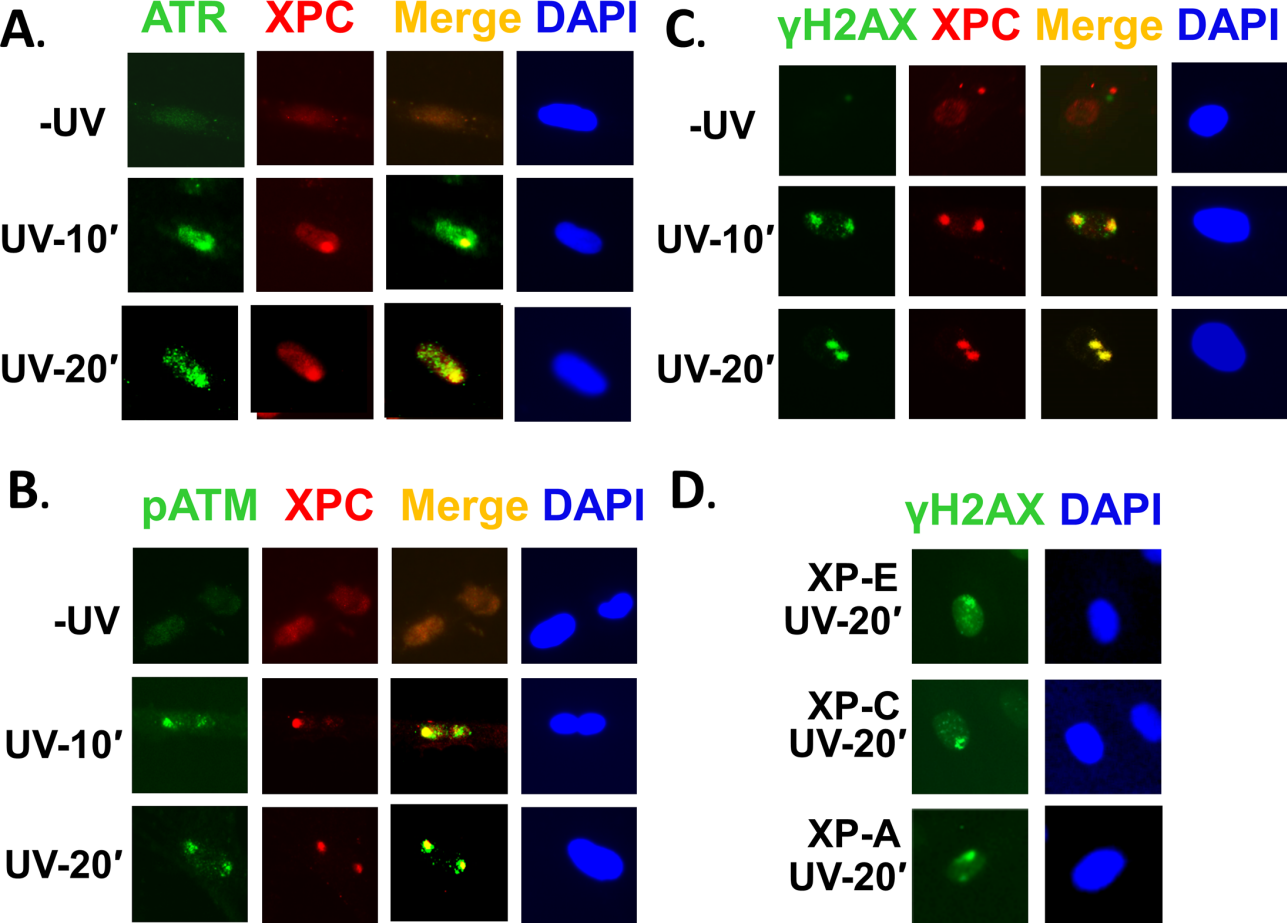
ATR and ATM recruitment and γH2AX phosphorylation at UVR damage sites in asynchronous OSU-2 cells. OSU-2 cells were exposed to 100 J/m^2^ UVR through a 5 μM micropore filter and allowed to respond to the induced damage for short indicated periods. The cells were fixed and immunofluorescence was performed to decorate ATR, pATM, γH2AX, and XPC proteins with cognate antibodies. Images show representative ATR, pATM and γH2AX foci co-localizing at DNA damage sites that were marked by distinct XPC labeling. (A) ATR (B) pATM (C) γH2AX (D) γH2AX foci formation in XP-E, XP-C, and XP-A cells upon 20 min post-repair.

To examine the timing of ATR and ATM recruitment to UVR-induced DNA damage sites, we performed a time course experiment to monitor the accumulation of ATR and ATM at the UVR lesion sites at different times following UVR exposures (data are only shown for initial 10 min and 20 min time points). We overlaid a micropore filter paper and exposed the cells at 100 J/m^2^ through the pores. In this method, wherever the UV radiation hits the nucleus in cells, all the involved protein factors accumulating at these sites can be easily detected by immunofluorescence [48]. We found that in asynchronous OSU-2 cells, both ATR and ATM are recruited as early as ten minutes and co-localize with XPC foci (Fig 1A & 1B). For detection of ATM, we used pATM antibody as ATM is phosphorylated only after its recruitment to the damage sites. Consequently, H2AX phosphorylation is also evident at the same ten minute post-UVR exposure time in OSU-2 cells (Fig 1C). These results indicate that ATR and ATM recruitment and H2AX phosphorylation start early upon UV irradiation and these proteins co-localize with early damage recognition factor XPC. We also examined the H2AX phosphorylation in repair-deficient XP-E (DDB2-mutated), XP-C (XPC mutated), and XP-A (XPA mutated) patient-derived fibroblast cell lines after 20 min post-repair. We observed that about 15–20% cells showed H2AX phosphorylation in XP-E, XP-C, and XP-A cells. In Fig 1D, a representative cell with γH2AX foci is shown for these cells (Fig 1D). The experiments suggest that these NER-deficient cells are equally proficient to form γH2AX foci at early times.

### ATR and ATM recruitment and their substrate phosphorylation are negatively affected in NER-deficient XP-A cells

**Fig 2:**
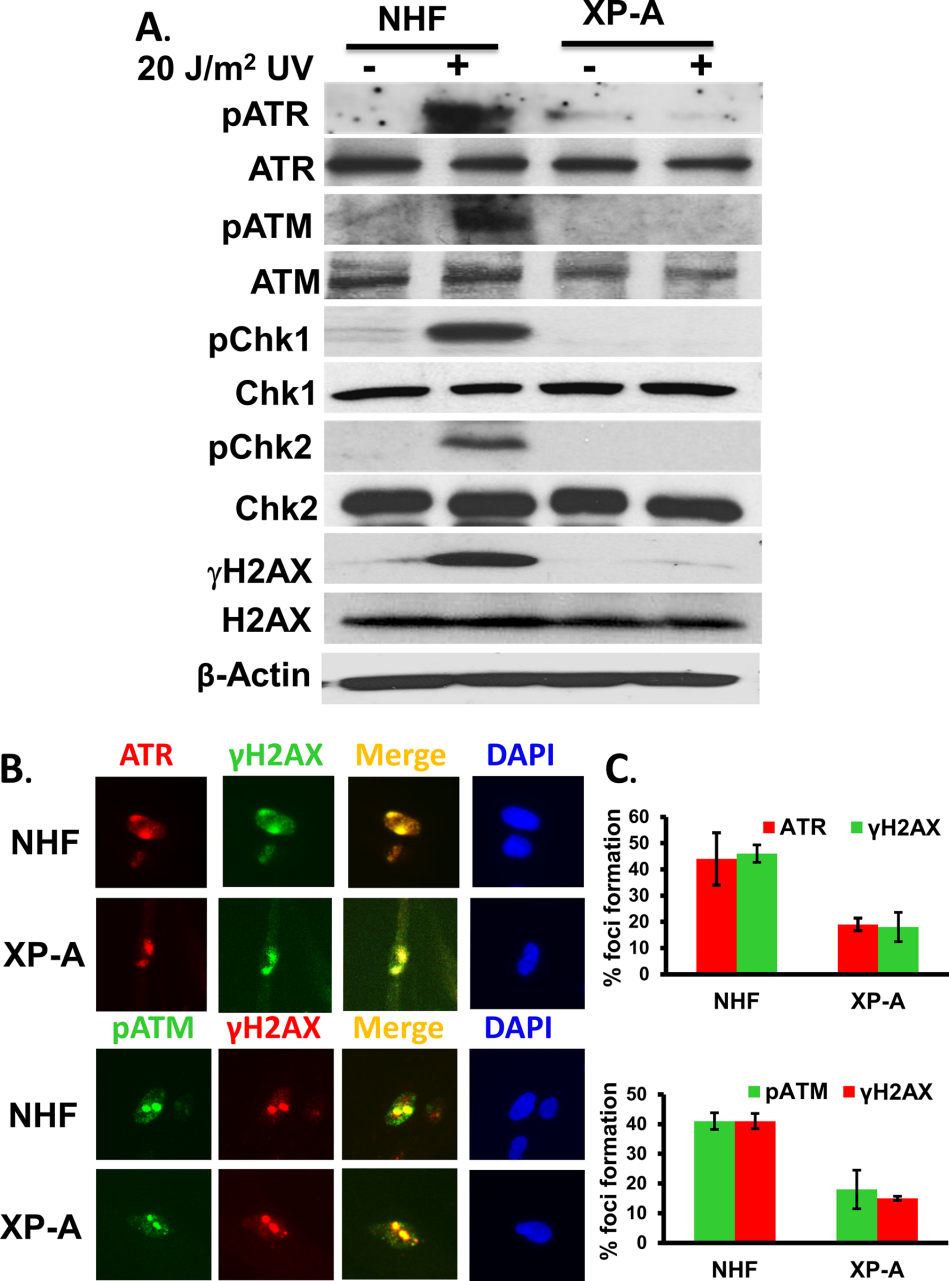
ATR, ATM and their substrate phosphorylations are affected in cells defective in XPA function. A. ATR and ATM and their substrate phosphorylation are affected in XP-A cells. Normal OSU-2 and XP-A cells were exposed to 20 J/m^2^ UVR and allowed to repair for 1 h. Their protein extracts were used to determine the phosphorylation of ATR, ATM, Chk1, Chk2, and H2AX alongside their total protein levels. Western blots were performed as described in [45]. (B) ATR and ATM recruitment and H2AX phosphorylation at the damage site is affected in XP-A cells. OSU-2 and XP-A cells were exposed to 100 J/m^2^ UVR and 1 h after cellular UVR exposures, cells were processed for immunofluorescent detection of damage-specific ATR, pATM, and γH2AX. Images show representative cells for individual treatment. The top panel shows the co-localization of ATR with the damage marker γH2AX, and the bottom panel shows the co-localization of pATM with γH2AX. (C) The quantitative data of ATR, pATM, and γH2AX foci formation: Quantitation was done by using three independent experiments, and at least 100 random samples per experiment were scored in each group. All values were expressed as mean ± SD. The difference between groups was tested using two-sided t-tests. The p-values <0.05 are considered significant.

Our previous studies established that GG-NER specific DDB2 and XPC influence ATR and ATM recruitment to the UVR-induced DNA damage sites [41]. However, XPA plays an essential role in both GG-NER and TC-NER and interacts with EXO1 to generate the ssDNA lesions at UV damage sites in G1 [49;50]. Furthermore, ATR was shown to interact with XPA upon UV irradiation [51] and phosphorylates XPA [52]. We observed that XP-A cells show about 17% of γH2AX foci upon 20 min post-repair, suggesting that ATR and ATM recruitment is affected in majority of the XP-A cells. Therefore, we wanted to examine if XPA is also involved in controlling ATR and ATM recruitment and their activation. To address this, we examined the phosphorylation of ATR and ATM and their downstream substrate proteins in repair-deficient XP-A cells and compared the signaling responses to that in repair-proficient OSU-2 cells. As shown in Fig 2A, the levels of ATR and ATM phosphorylation were significantly reduced in XP-A cells as compared to OSU-2 cells, indicating that XPA is required for ATR and ATM recruitment. As a result, ATR and ATM substrate Chk1 and Chk2 phosphorylations were also dramatically affected in cells defective in XP-A function (Fig 2A). We further observed that even though ATR, pATM, and γH2AX foci could be seen in a fraction of XP-A cells, quantitatively there was a substantial reduction in frequency of cells with positive foci in XP-A compared to OSU-2 cells (Fig 2B & 2C). When ATR and γH2AX co-localization was performed, ATR foci were present in 19% and γH2AX foci were present in 18% of XP-A cells as compared to 44% and 46% in OSU-2 cells, respectively (Fig 2C, top panel). Similarly, we observed that pATM foci were 18% and γH2AX foci were 15% in XP-A cells as compared to 41% each in OSU-2 cells (Fig 2C, bottom panel). In the immunofluorescence figure, only the representative cells containing the positive foci are shown (Fig 2B), while the cells lacking foci are accounted through quantitative assessment of all cells with and without foci (Fig 2C). These results showed that ATR and ATM recruitment and activation by phosphorylation at the UV damage sites are negatively affected in majority of the XP-A cells, indicating that ATR and ATM recruitment is influenced by the association of XPA at the UVR damage sites.

### ATR and ATM recruitment and activation at UVR-induced damage sites depend on DDB2, XPC, and XPA during G1 phase of cell cycle

**Fig 3:**
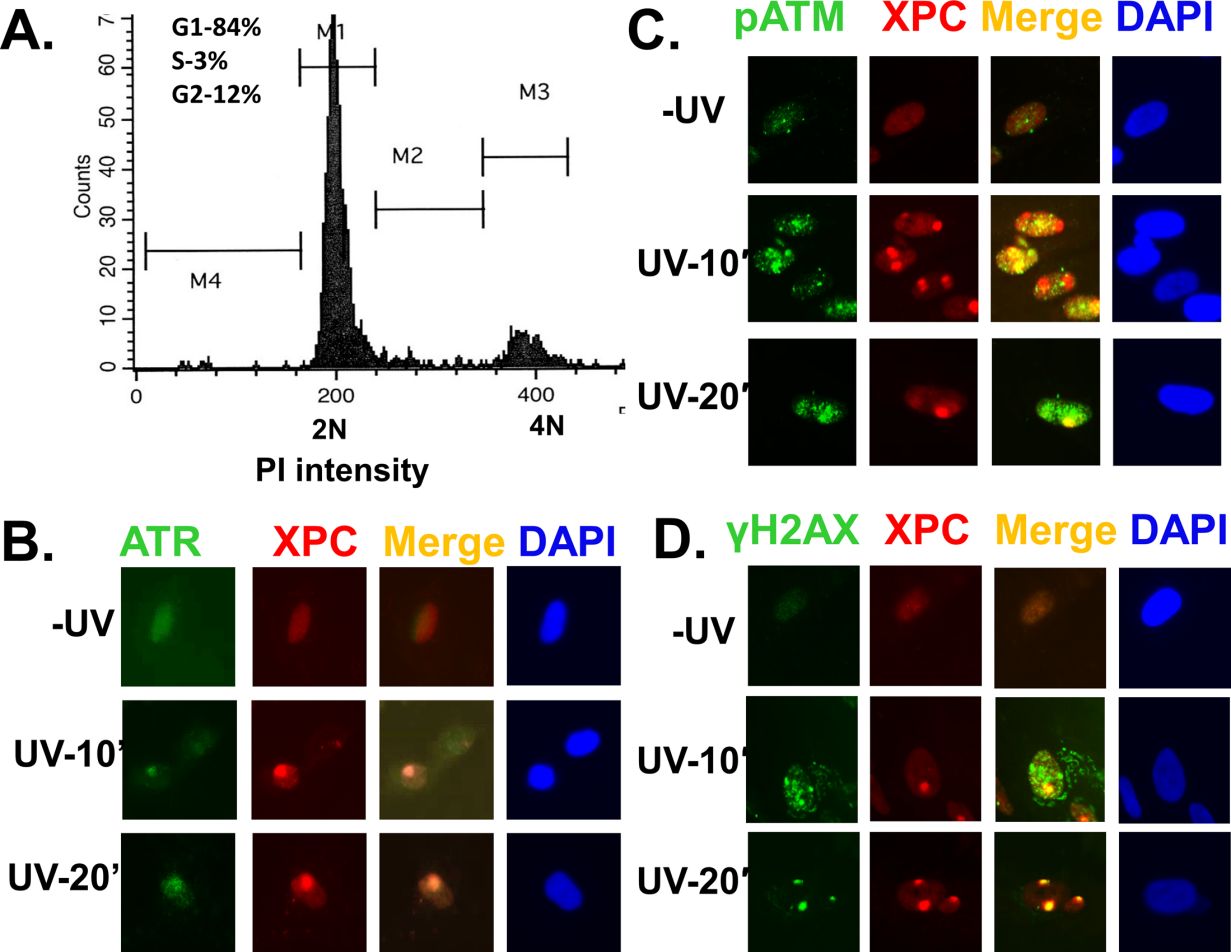
ATR and ATM recruitment and H2AX phosphorylation at UVR damage site in G1 cells. A. OSU-2 cells were arrested by in G1 phase of cell cycle by serum starvation for 48 h and the cell cycle arrest was examined by FACS analysis. B, C, &D. Experiments were done as described in Fig 1 using the G1 arrested cells.

**Fig 4:**
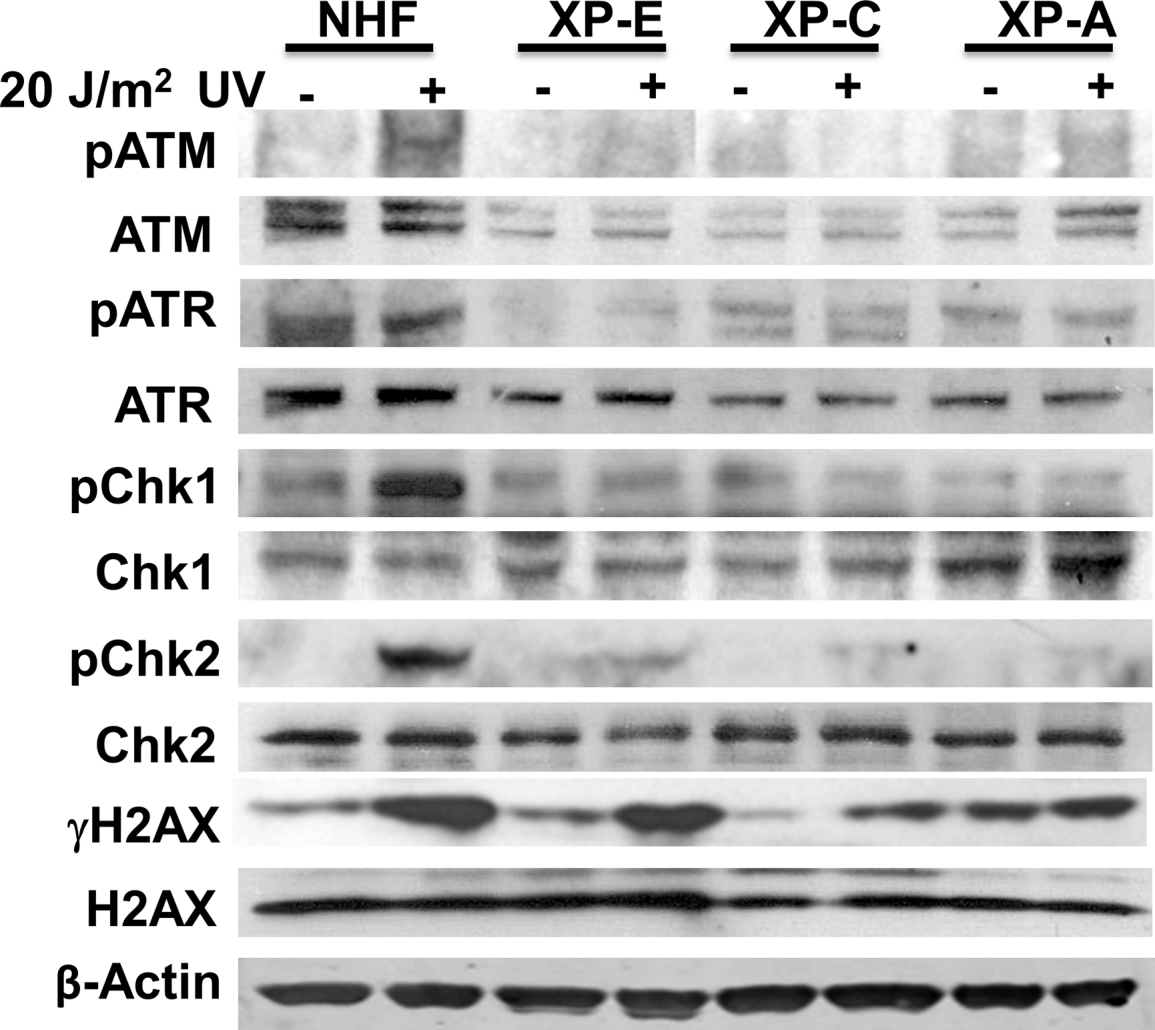
ATR and ATM and their substrate phosphorylation are affected in G1 arrested cells. OSU-2, XP-E, XP-C and XP-A cells were arrested in G1 phase of cell cycle as described in A, exposed to 20 J/m^2^ UV irradiation, and after 1 h post-repair time, extracts were isolated and processed by Western blotting as described in [45].

**Fig 5:**
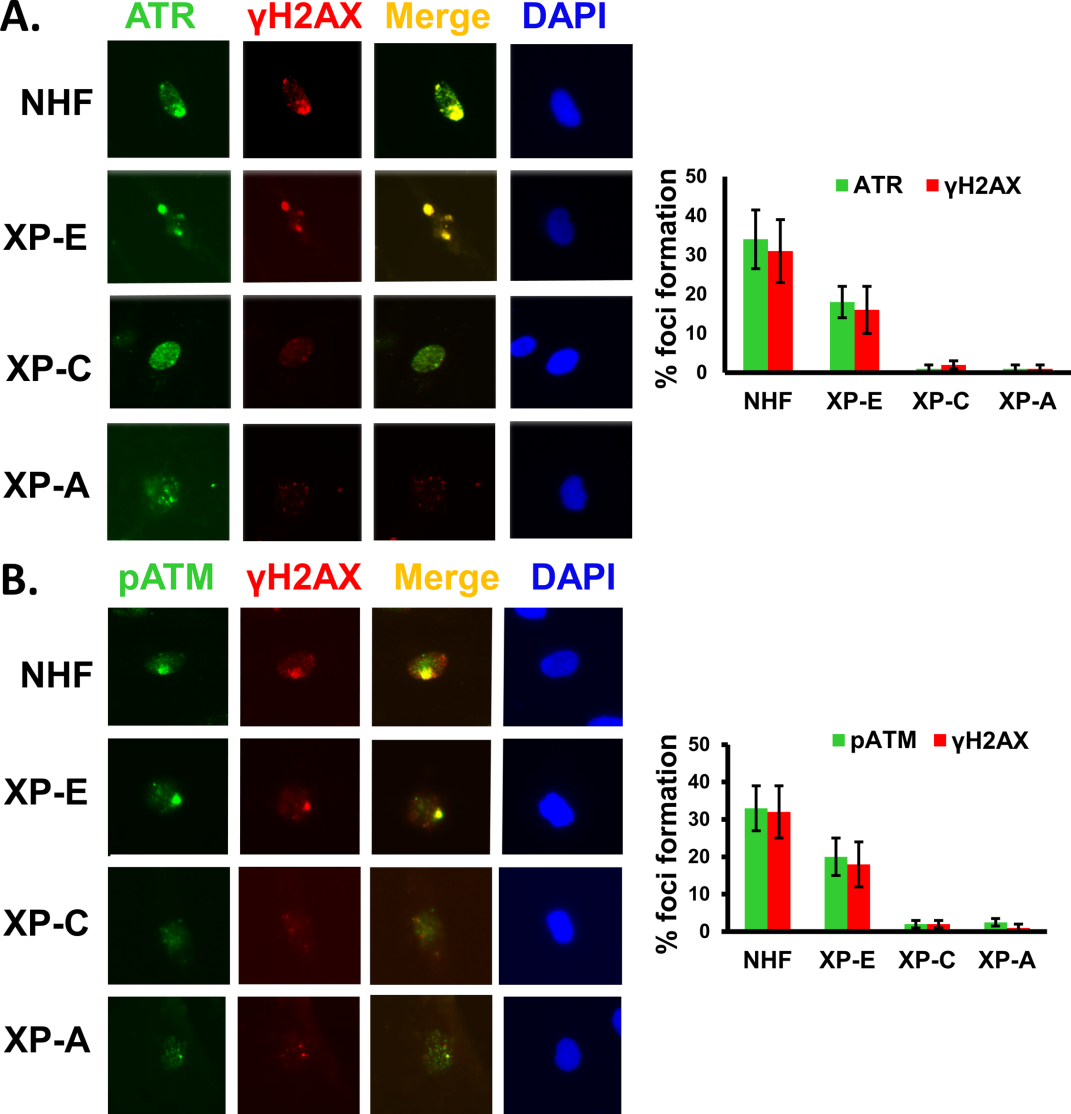
ATR, ATM recruitment and H2AX phosphorylation is affected in XP cells during G1. (A) Colocalization of ATR with DNA damage marker γH2AX in G1 arrested cells. OSU-2, XP-E, XP-C and XP-A cells were arrested in G1 phase of cell cycle by serum starvation for 48 h and exposed to 100 J/m^2^ UV irradiation through a 5 μm micropore filter. At 1 h post-exposure immunofluorescence was performed to determine the extent of co-localization. The quantitative data of ATR and γH2AX foci formation were determined as described in Fig 2B (B) Colocalization of pATM with γH2AX in G1 arrested cells. Experiments were done as described in A.

**Fig 6:**
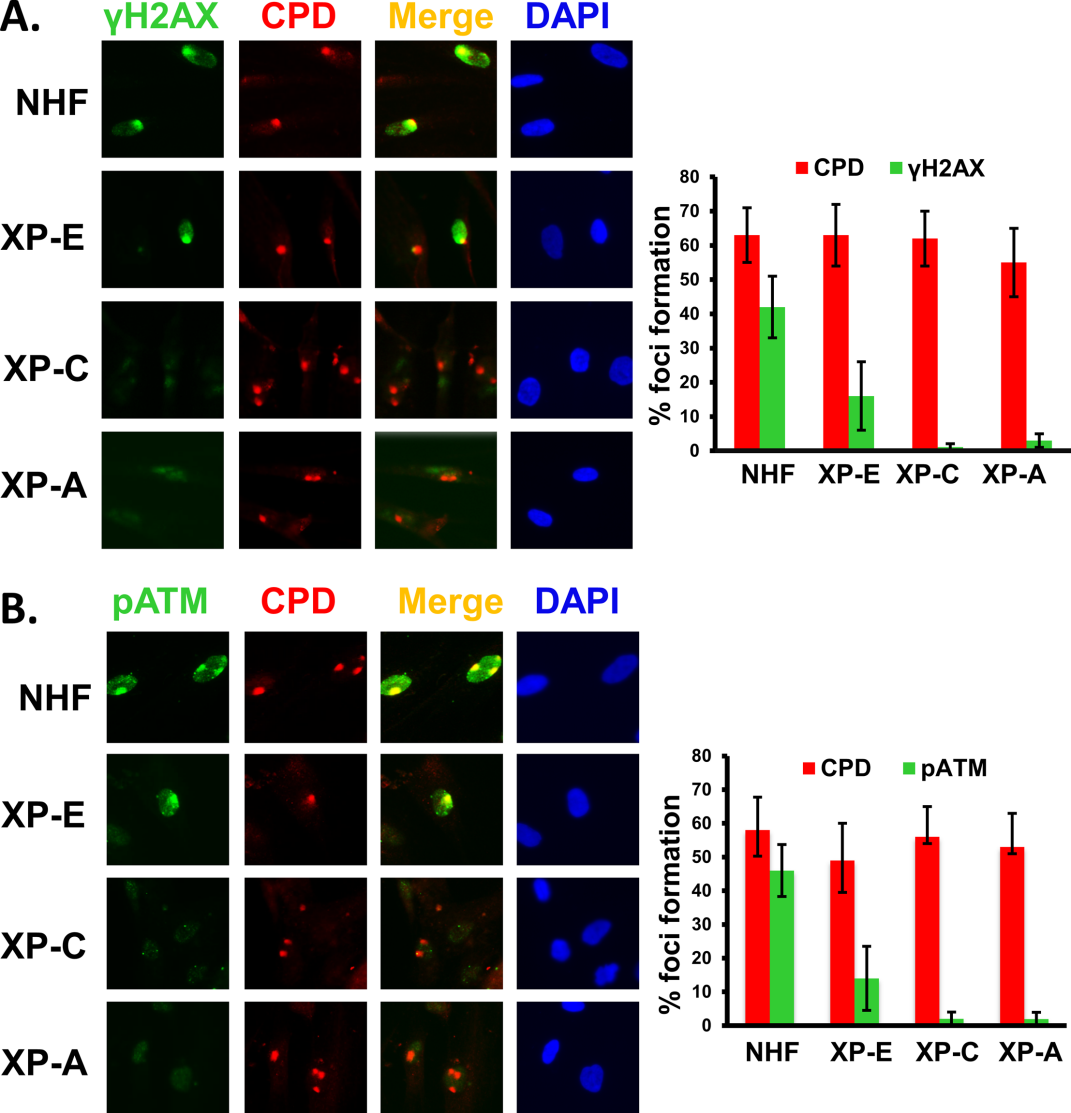
ATM recruitment and H2AX phosphorylation at damage sites is affected in XP cells during G1. (A) γH2AX foci formation at the CPD damage sites. OSU-2, XP-E, XP-C and XP-A cells were arrested in G1 by serum starvation for 48 h and exposed to 100 J/m^2^ UV irradiation using a 5 μm micropore filter. After 1 h post-exposure time immunofluorescence was performed as in Fig 5. The quantitative data of γH2AX and pATM foci formation were determined as described in Fig 2B. (B) pATM foci formation at the CPD damage sites. Experiments were done as described in A.

Our experiments using asynchronous cells showed that ATR and ATM recruitment is partially influenced by DDB2, XPC, and XPA [41] (Fig 2). This raised the possibility that the dependence of ATR and ATM recruitment on NER proteins might be dependent on the cell cycle phases and different lesions associated with the cell cycle. To test this, we first determined the recruitment of ATR and ATM at the UVR damage sites in G1 arrested OSU-2 cells by immunofluorescent visualization of cognate factor recruitment. Cells were arrested by serum starvation for 48 h and propidium stained cells analyzed by FACS to examine the G1 arrest. About 85% cells were shown to arrest in G1 by this method (Fig 3A). We found that both ATR and ATM are promptly recruited to UVR damage sites in G1 arrested OSU-2 cells as early as 10 min, and the foci intensified further at 20 min time indicating an increasing congregation of these proteins at the UVR damage sites with elapsing time (Fig 3B & 3C). We did not see further increase in foci intensity beyond 30 min of post-repair time (data not shown). Accordingly, H2AX phosphorylation in discrete foci was also evident as early as 10 min in these cells (Fig 3D). Next, we investigated if DDB2, XPC, and XPA influence ATR and ATM function in G1 arrested cells. For this, we examined the levels of ATR and ATM phosphorylation as well as their downstream substrates phosphorylation in G1 phase of cell cycle upon UV irradiation. The effects were determined in G1 arrested repair-proficient OSU-2 cells and compared with G1 arrested repair-deficient XP-E, XP-C, and XP-A cells. Using these G1 arrested cells, we demonstrated that ATR and ATM were activated by phosphorylation in OSU-2 cells, as evident by the levels of phosphorylated ATR and ATM (Fig 4A, lane 1 and 2). As a result, ATR and ATM substrates Chk1 and Chk2 respectively, were also phosphorylated in OSU-2 cells. Consequently, ATR- and ATM-mediated H2AX phosphorylation was also reduced in XP-A cells. In contrast, ATR and ATM as well as the target substrate phosphorylation were drastically impaired in all repair-deficient G1 arrested cells (Fig 4A, lanes 3–8). These results support that upon UV irradiation, the NER proteins influence ATR and ATM activation and their substrates’ phosphorylation in G1 arrested cells. We also determined the ATR, ATM, and γH2AX foci formation in G1 arrested cells and quantitated the effects exerted by the presence and absence of NER factors on the accumulation of ATR, ATM, and γH2AX at damage sites. The DDB2-deficient XP-E cells, which show milder NER-defect phenotype, exhibited a relatively small but quantitatively significant reduction in the level of these phosphorylation events. For example, XP-E cells showed 18% ATR, and 16% γH2AX co-localizing foci containing cells compared to 34% and 31% in OSU-2 cells, respectively (Fig 5A). In similar experiments, XPE cells exhibited 20% pATM, and 18% γH2AX co-localizing foci containing cells compared to 33% and 32% in OSU-2 cells, respectively (Fig 5B). Importantly, however, ATR and ATM recruitment and the consequent H2AX phosphorylation was almost completely abrogated in G1 arrested repair-deficient XP-C and XP-A cells. We also confirmed these results by showing that pATM recruitment and H2AX phosphorylation occurred at DNA damage sites marked by authentic CPD lesions induced at the UVR exposed sub-nuclear spots (Fig 6A & 6B). Here, we detected CPD foci in about 50–60% cells in both repair-proficient and -deficient human cells. However, as observed in Fig 5, both pATM and γH2AX foci were completely abrogated in G1 arrested XP-C and XP-A cells (Fig 6A & 6B). As expected, and as seen in Fig 5, G1 arrested XP-E cells showed a relatively lower reduction in the frequency of CPD co-localizing γH2AX and pATM foci, i.e., 16% γH2AX and 14% pATM foci in XPE cells as compared to 42% γH2AX and 46% pATM foci in OSU-2 cells. In repair-deficient cells, the frequency of CPD foci remained unaffected but the corresponding damage response phosphorylation events were undetectable. Taken together, these studies demonstrated that in G1 phase of cell cycle, ATR and ATM recruitment and H2AX phosphorylation are regulated by DDB2, XPC, and XPA proteins. Our quantitative analysis revealed that ATR, ATM, and H2AX phosphorylation is moderately affected in XP-E cells, but completely abrogated in XP-C and XP-A cells. This is consistent with the fact that, in executing NER, DDB2 is needed for CPD repair, but not for 6-4PP repair whereas XPC and XPA are needed for both CPD and 6-4PP repair [3;6;8]. Thus, even in the absence of DDB2, sufficient XPC protein congregates at the UVR lesions to positively affect the recruitment of ATR and ATM to the damaged foci, and allow the corresponding H2AX phosphorylation.

### H2AX phosphorylation and ATM recruitment upon UV irradiation do not depend on DDB2, XPC, and XPA in S phase of cell cycle

**Fig 7:**
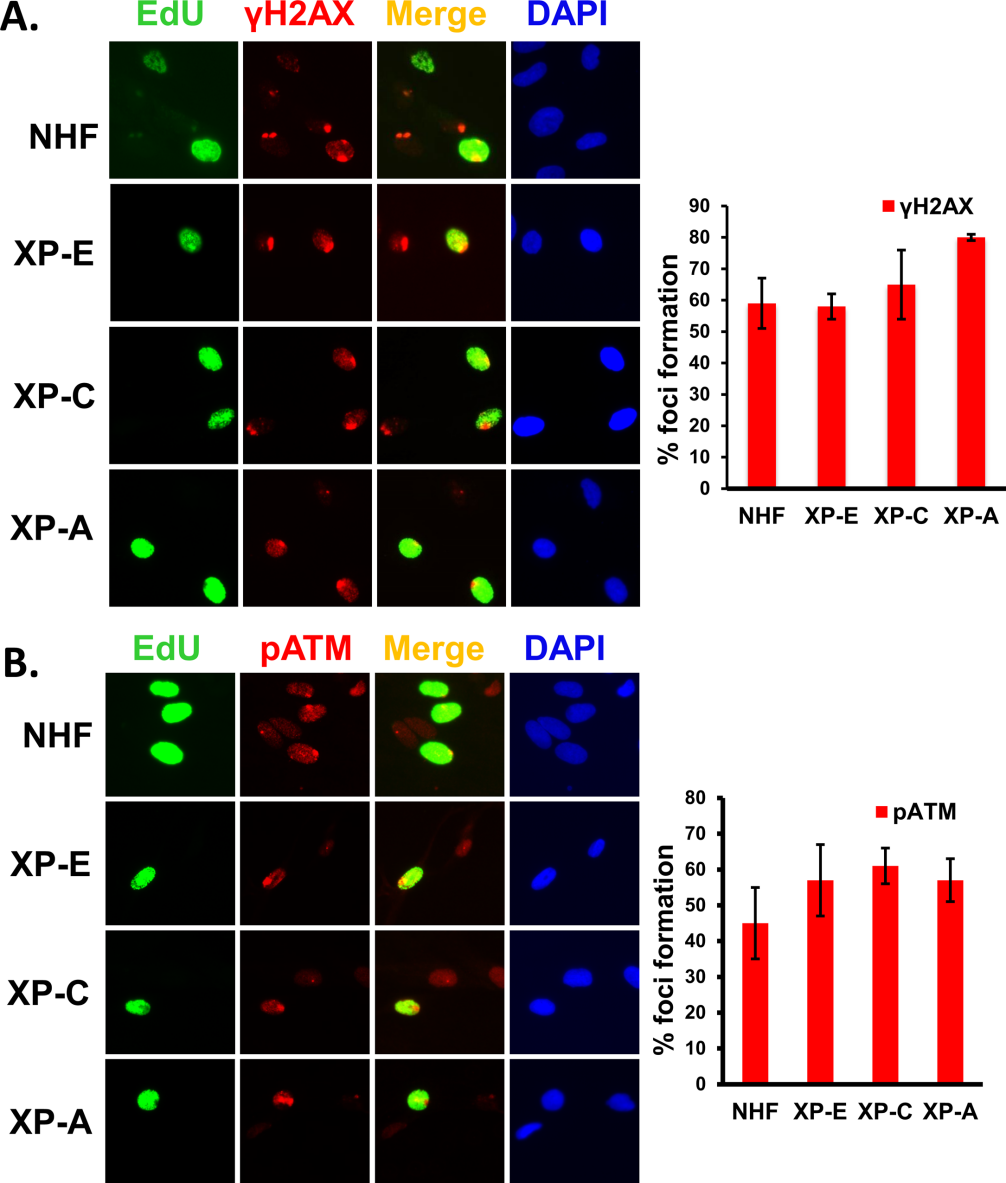
ATM recruitment and H2AX phosphorylation at damage sites is not affected in XP cells during S. (A) γH2AX foci formation in EdU positive cells. Asynchronously growing OSU-2, XP-E, XP-C and XP-A cells were exposed to 100 J/m^2^ UVR through a 5 μm micropore filter. S phase cells were differentiated from non-S phase cells by nuclear EdU labeling as described in methods. The H2AX was detected by immunofluorescence as described in Fig 1. The quantitative data of γH2AX and pATM foci formation in S phase EdU-positive cells were determined as described in Fig 2B. (B) pATM foci formation in EdU positive cells. Experiments were done as described in A.

During S phase of cell cycle, progression of replication forks stall when it encounters UVR-induced CPD, 6-4PP, or ssDNA gaps due to NER processing [53]. Upon stalling, the uncoupling of replicative helicases and polymerases at blocked forks leads to long stretches of ssDNA, and this ssDNA is recognized by ATRIP-RPA which helps in the recruitment of ATR. Activation of ATR and Chk1 can stabilize stalled forks and prevent fork collapse [18]. Despras et. al. showed that S phase cells have higher levels of ssDNA which co-localizes with RPA and PCNA, and these ssDNA stretches are the sites of H2AX phosphorylation by ATR [54]. Ward and Chen have shown that replication stress due to UVR induces ATR-dependent H2AX phosphorylation at the replication damage sites during S phase of cell cycle [55;56]. To assess the underlying role of ATR and ATM pathway in the regulation of H2AX phosphorylation during S phase, we first examined the levels of H2AX foci formation in S phase cells upon UVR-induced replication stress. By using repair-proficient OSU-2 and repair -deficient XP-E, XP-C, and XP-A cells, we investigated whether H2AX phosphorylation is affected in different repair-deficient cells during S phase of cell cycle. We used ethynyl deoxyuridine (EdU) labeling approach to distinguish the S phase and non-S phase cells within the mixed population of asynchronously growing cells [46;47]. We found that EdU staining reveals bright and homogenously distributed fluorescence only in the nuclei of S phase cells (Fig 7). An examination of the non-S phase cells, which are DAPI-positive without any green fluorescence (Edu-negative), show a conspicuous lack of foci formation in repair-deficient XP-C and XP-A cells, corroborating the results described above for G1-arresed cells. Here, however, the evaluation of damage response was focused on EdU-marked S phase cells which exhibited a clear and strong staining of γH2AX foci. The data reveal that EdU-positive S phase cells harbor distinct DNA damage foci in repair-proficient and especially in repair-deficient cells. More importantly, the frequency of γH2AX foci was essentially comparable in all cell types as no significant reduction of foci formation could be observed in repair-deficient or repair-proficient cells (Fig 7A). In fact, a slight increase of γH2AX foci formation could be seen in repair-deficient XP-C and XP-A cells. For example, in XP-E, XP-C, and XP-A cells, γH2AX foci frequency were 58%, 65%, and 80%, respectively, as compared to 59% in OSU-2 cells. Therefore, it could be concluded that H2AX phosphorylation in S phase cells is not influenced by the NER complex and the increase in γH2AX foci of XP-C and XP-A cells might indicate the presence of a higher extent of unprocessed lesions due to their inability to repair UVR-induced DNA damage.

If the stalled forks collapse, they create DSBs which specifically recruit MRN complex and ATM. These proteins allow effective strand invasion of the sister chromatid for the reestablishment of the replication fork. To assess if these processes are regulated by the NER proteins, we again performed the localization of ATM in EdU-positive S phase cells. Here, quantitation in XP-E, XP-C, and XP-A cells showed 57%, 61%, and 57% pATM foci, respectively, as compared to 45% of pATM foci in OSU-2 cells. Similar to γH2AX foci formation, these results also indicated a small but discernable increase in the frequency of pATM foci in the repair-deficient cells (Fig 7B). Thus, these results reinforce the conclusion that the UVR-induced activation of damage response, observed through ATM recruitment in S phase cells, does not depend on the participation of NER proteins at DNA damage sites.

## Discussion

We have previously demonstrated that ATR and ATM recruitment is dependent on the assembly of functional DNA repair proteins, and in the present study we have now established that the UVR-mediated ATR and ATM recruitment occurs as a very early event in asynchronous and G1 arrested cells (Figs 1 & 3). DDB2 and XPC have the inherent recognition capacity for UVR-induced DNA damage, and instigate the prompt formation of pre-incision complex at the damage sites [5;57–59]. DDB2 and XPC recruitment can be immuno-detected at the damage sites by ~5 minutes post-irradiation [57;60]. An early ATR and ATM recruitment established the timing of their recruitment and supports the contribution of both ATR and ATM in early H2AX phosphorylation and checkpoint signaling.

To demonstrate DDR upon UV damage, most of the previous studies focused on the detection of H2AX phosphorylation, which is ATR and ATM substrate and did not explore the mechanism of ATR and ATM recruitment to the damage sites [33;50;61]. Our previous study demonstrated that ATR and ATM physically and functionally interact with XPC. Additionally, DDB2 and XPC regulate ATR and ATM recruitment and signaling following UVR exposures [41]. Here, we further demonstrated that both ATR and ATM recruitment and activation is affected by another core NER factor, XPA. These new results suggest that ATR and ATM recruitment requires a functional NER apparatus that includes DDB2, XPC, and XPA for invoking the critical signaling events. These results are consistent with observations of Sertic et. al., demonstrating that hEXO1 interacts with XPA and connects NER processing with checkpoint activation in response to UV irradiation [46].

**Fig 8:**
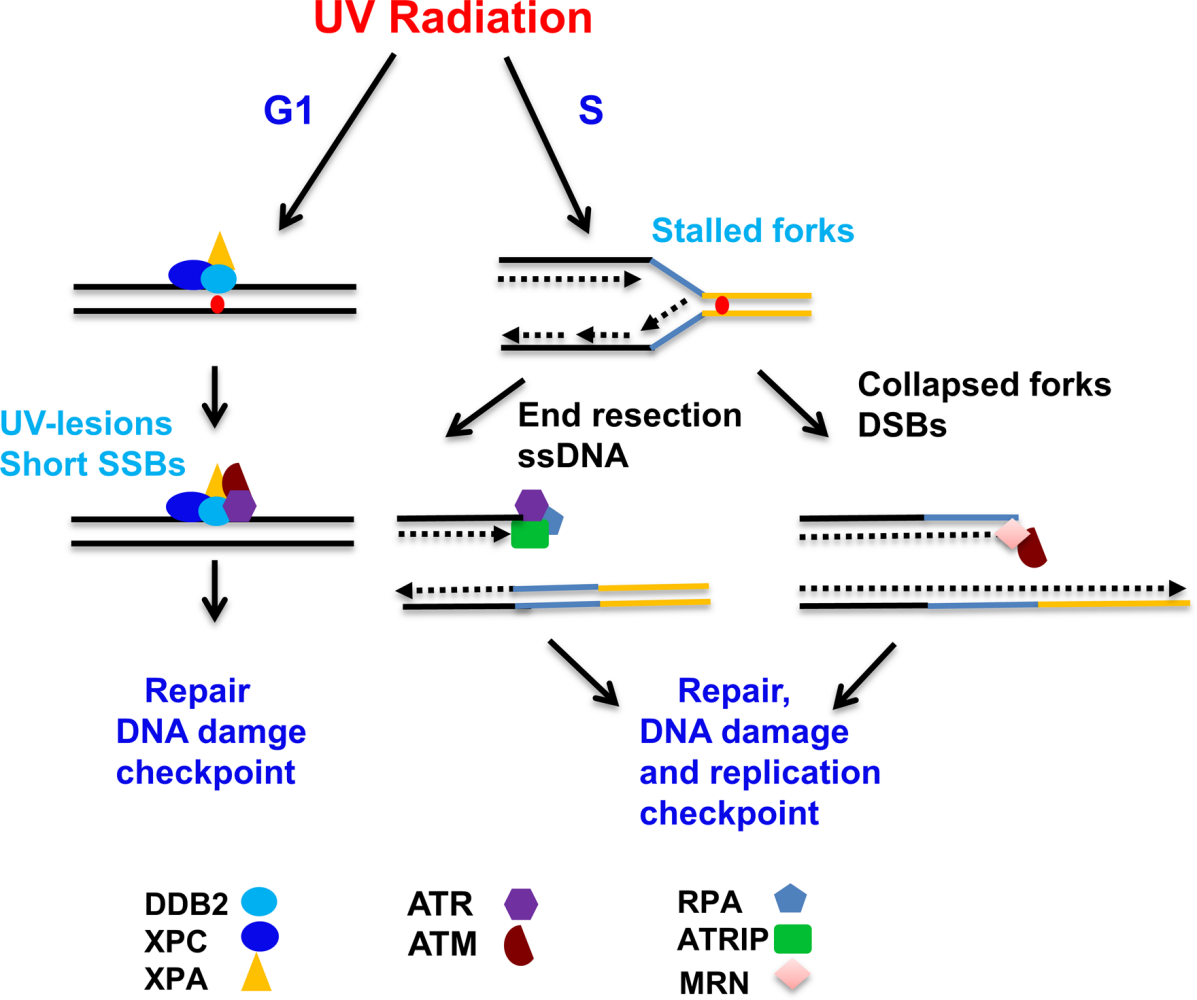
Schematic model of pre-incision and pre-checkpoint UVR response events. Only the predominant pathways involved in post-UVR regulation of ATR and ATM recruitment during G1 and S phase of cell cycle are considered. G1 phase cells possess UVR-induced lesions which primarily recruit core components of NER pathway. Formation of the NER complex in turn facilitates the recruitment of ATR and ATM to initiate repair and in parallel the downstream events leading to G1 checkpoint. In S phase cells, replication fork encounters the UVR-induced lesions which result in the formation of ssDNA breaks and recruitment of ATR to processed single strand ends. Replication fork collapse results in DSBs where ATM is recruited. Both of these SSBs and DSBs lead to the initiation of S phase replication checkpoint and are repaired by HR pathway.

Our results of ATR and ATM recruitment to the UVR damage sites showing that ATR and ATM localization as well as H2AX phosphorylation are regulated by NER proteins during G1, but not in S phase of cell cycle are quite intriguing. Previous studies in this area showed that H2AX phosphorylation upon UV radiation is triggered by both NER-dependent and independent pathways [50;61–63]. Although some of these studies were undertaken with G1 and S arrested cells, they examined H2AX phosphorylation, and none of them investigated recruitment of ATR and ATM in a cell cycle specific manner. To resolve the contradiction, it was necessary to revisit the concepts and hypothesis by analyzing not only γH2AX foci formation, but also revealing the status of the kinases which phosphorylate H2AX and other checkpoint kinases, e.g., Chk1 and Chk2. Therefore, we used the upstream kinases along with H2AX phosphorylation to dissect the cell cycle specific processes. Our results demonstrated that assembly of ATR-Chk1 and ATM-Chk2 complex at the UVR-lesions are regulated by NER complex in G1 at a very early time. This data is supported by Marini et. al. showing that UV-induced Chk1 phosphorylation is restricted in G_1_ and G_2_/M phases in XP-A mutated cells, but not in S phase cells [50]. Additionally, Sertic et. al. also found that hEXO1 association to NER processed ssDNA is restricted to non-replicating cells, and is needed for checkpoint activation in G1 [46]. Therefore, we reckon that ATR and ATM associate with XPA-hEXO1 complex exclusively during G1 phase to influence repair and checkpoint. We clearly demonstrate that ATM is also an important regulator of UV damage response in G1 phase, in contravention of the earlier view supporting the exclusive role of ATR, in the UVR-mediated signaling and H2AX phosphorylation in G1 phase [32;33;62]. Furthermore, our data clearly demonstrated that ATM is also needed for early damage response in G1 phase. Indeed, recent studies by Wakasugi *et*. *al*. showed that NER-dependent DSBs in quiescent G_0_ cells activate ATM kinase and trigger H2AX phosphorylation upon UVR exposure [64]. Previous studies showed that in G1, checkpoint activation is due to the ssDNA and not from DSBs [33;46;49] and therefore it is highly likely that ATR/ATM is recruited to the NER processed long ssDNA where EXO1 binds [46]. Our results showing the presence of similar levels of phosphorylated H2AX and ATM in repair-deficient cells compared to repair-proficient cells suggests that NER complex is not necessary for ATR and ATM recruitment to the S phase lesions. This is supported by Marini et. al., who showed that Chk1 and p53 phosphorylation are not dependent on DDB2, XPC, and XPA in S phase cells [50]. However, Bomgarden et. al. showed that even though ATRIP localization and Chk1 phosphorylation is not affected in XP-C, XP-F, and XP-G cells, it is affected in XP-A cells [65]. This discrepancy might be due to the experimental procedure, cell lines, and UV dose of 50 J/m^2^ instead of 20 J/m^2^. The S phase cells predominantly possess long stretches of ssDNA at stalled replication forks in front of the UVR-induced photoproducts which recruit ATR. Upon recruitment, ATR phosphorylates H2AX near the stalled replication forks. Collapsing of the replication forks generates DSBs. DSB recruits MRN complex, which subsequently facilitates ATM recruitment, H2AX phosphorylation, and DNA repair by HR pathway [20;21;40]. Accordingly, upon UV irradiation and replication fork stalling, replication repair factors such as BRCA1, RAD51 and FANCD2 form nuclear foci [34]. Based on these previous S phase damage response studies, it is very likely that recruitment of ATR and ATM is independent of NER factors in S phase. Collectively, our data unambiguously show that ATM and ATR recruitment and H2AX phosphorylation during G1 phase is dependent on NER proteins, but independent of NER proteins in S phase. Based on our results, we propose a model showing different types of DNA lesions generated during G1 and S phases, and how they would influence recruitment of proteins for repair and induction of cell cycle checkpoints (Fig 8). We cannot rule out the possibility that during S phase, DDB2, XPC, and XPA also regulate ATR and ATM recruitment to some of the UVR lesions generated by NER pathway at the non-replicating regions, which are not encountered by stalled forks. However, detection of such H2AX and ATM phosphorylation in repair-deficient cells would be difficult due to the abundance of signal emanating from the stalled replication sites. Nevertheless, our data is supported by recent results of Quinet *et*. *al*., which show that XP-C cells despite the accumulation of ssDNA do not exhibit an apparent defect in S phase progression whereas Polη-deficiency resulted in S phase arrest [66]. It is difficult to define the types of lesions where ATR and ATM are recruited within the cell cycle, but our approach using single-cell analysis enabled us to show that defects in DDB2, XPC, and XPA do interfere with UV-induced ATR and ATM recruitment in G1, but not in S phase of cell cycle.

Considering the fact that structurally different lesions are repaired by various mechanisms and in a cell cycle specific manner, understanding the mechanistic aspects influencing repair efficiency is highly relevant for defining the basis of genome instability and oncogenesis. Moreover, the NER-mediated secondary DNA damage, such as ssDNA and DSBs can be formed in senescent cells in our body causing a serious problem as majority of our cells undergo senescence. Therefore, the Xeroderma pigmentosum patients deficient in DDB2, XPC, and XPA genes are highly cancer prone and display over 2,000-fold increased incidence rates of skin cancer due to defects in NER pathway. The skin cancer rate is increasing in the US every year, more than breast, prostate, lung, and colon cancer combined; therefore understanding the signaling pathways that determine UV sensitivity is important to reduce the high mortality rate in skin cancer [67;68]. Furthermore, Heterozygosity for XP is also a high risk factor for several cancers [67–70]. Defects in ATR-Chk1 and ATM-Chk2 signal transduction pathways are also key feature of several human cancers. ATM, ATR, Chk1, Chk2 have been identified as candidate multi-organ tumor suppressor genes and their mutations lead to oncogenic transformations [14;71;72]. ATR has already been shown to protect from cancers. Notably, studies by Wakasugi et. al. showed that ATM-deficient cells exhibited enhanced UV sensitivity, and Hannan et. al. showed that ATM-deficient cells show cell death upon UVR exposure [73], showing that ATM plays a significant role in UV-induced carcinogenesis. Furthermore, recent study by Li et. al., showed that both ATR and ATM are highly mutated (30–35%) in cutaneous squamous cell carcinoma which is mostly associated with UV exposure [74]. The processing of DNA damage, cross-talk between the repair and checkpoint factors, and regulatory mechanisms of their recruitment during specific phases of cell cycle are necessary for complete understanding of DNA damage response and its contributory role in tumorigenesis.
